# Trends in Cause-Specific Outcomes Among Individuals With Type 2 Diabetes and Heart Failure in the United Kingdom, 1998-2017

**DOI:** 10.1001/jamanetworkopen.2019.16447

**Published:** 2019-12-02

**Authors:** Claire A. Lawson, Francesco Zaccardi, Gerry P. McCann, Melanie J. Davies, Umesh T. Kadam, Kamlesh Khunti

**Affiliations:** 1 Diabetes Research Centre, University of Leicester, Leicester, United Kingdom; 2 Department of Cardiovascular Sciences, University of Leicester, Leicester, United Kingdom; 3 Leicester NIHR Biomedical Research Centre, Leicester, United Kingdom; 4 Department of Health Sciences, University of Leicester, Leicester, United Kingdom

## Abstract

**Importance:**

The phenotype of individuals with type 2 diabetes and heart failure (HF) is changing. Successful public health interventions for type 2 diabetes mean that patients more frequently present with HF without a prior ischemic event, which is likely to change outcomes, but trends in cause-specific outcomes are unknown.

**Objective:**

To investigate cause-specific outcomes and trends associated with type 2 diabetes among individuals with incident HF.

**Design, Setting, and Participants:**

This cohort study used UK primary care data, linked to hospital admissions and mortality, for 87 709 patients with incident HF from 1998 to 2017. Patients were 30 years or older and observed to death or July 31, 2017. Data analysis was conducted in March and April 2019.

**Exposure:**

Preexisting type 2 diabetes at diagnosis of HF. Individuals with type 1 diabetes were excluded.

**Main Outcomes and Measures:**

All-cause, cardiovascular (CVD), and non-CVD unplanned hospitalizations and mortality rates.

**Results:**

Of 87 709 patients with HF (43 173 [49.2%] women; 78 211 [89.2%] white), 20 858 (23.8%) had type 2 diabetes (median [interquartile range] age, 78.0 [70.0 to 84.0] years), and 66 851 (76.2%) had no diabetes (median [interquartile range] age, 80.0 [72.0 to 86.0] years). In patients with HF, type 2 diabetes was associated with an increase in the risk of unplanned hospital admission (adjusted incidence rate ratio for CVD hospitalizations: 1.24; 95% CI, 1.19 to 1.30; for non-CVD hospitalizations: 1.26; 95% CI, 1.22 to 1.30) and an increase in the risk of mortality (adjusted hazard ratio for CVD mortality: 1.06; 95% CI, 1.02 to 1.10; for non-CVD mortality: 1.24; 95% CI, 1.19 to 1.29). Age-standardized mortality risk at 1 year was 35.6% (95% CI, 35.1% to 36.1%) in the type 2 diabetes group vs 29.2% (95% CI, 29.0% to 29.5%) in the group with no diabetes. During the study period (ie, 1998 to 2017), associations of type 2 diabetes with hospitalization and mortality rates decreased for CVD outcomes but not for non-CVD outcomes. Age-adjusted hospitalization rates during the first year following HF diagnosis increased similarly for both groups over time (eg, HF with type 2 diabetes, 1998 to 2001: 133.3 per 100 person-years; 95% CI, 102.2 to 105.4 per 100 person-years; 2012 to 2015: 152.5 per 100 person-years; 95% CI, 145.5 to 159.5 per 100 person-years; *P* for difference in trend = .06), but trends diverged by cause. For example, hospitalizations for HF decreased for patients with type 2 diabetes at approximately the same annual rate (−2.2%; 95% CI, −3.9% to −0.5%) as they increased for those without diabetes (1.7%; 95% CI, 1.1% to 2.3%; *P* for difference in trend < .001). After 2004, a trend emerged showing a greater increase in non-CVD admissions among patients with HF and type 2 diabetes than among patients with no diabetes (2.3% [95% CI, 0.9% to 3.6%] vs 1.1% [95% CI, 0.8% to 1.4%]). In contrast to hospitalization rates, mortality rates reduced over time in both groups, but the reduction was greater among those with type 2 diabetes than without (−1.4% [95% CI, −1.8% to −0.9%] vs −0.7% [95% CI, −1.2% to −0.2%]; *P* for difference in trend < .001).

**Conclusions and Relevance:**

In this study, the higher risk of all cause-specific outcomes and emerging non-CVD trends associated with patients with type 2 diabetes who experienced HF indicated an urgent need for earlier comorbidity management and patient-centered multimorbidity care.

## Key Points

**Question:**

Among the general population of individuals with type 2 diabetes who experienced heart failure, have cause-specific outcomes (ie, hospitalization and mortality) changed between 1998 and 2017?

**Findings:**

In this cohort study of 87 709 people with incident heart failure, type 2 diabetes was associated with significantly higher rates of cardiovascular disease (CVD)–related hospitalizations, non-CVD hospitalizations, and death. Cardiovascular disease risk associated with type 2 diabetes reduced significantly over the 20-year period, but non-CVD risk persisted, and non-CVD hospitalization rates among patients with heart failure and type 2 diabetes increased more quickly than among patients without diabetes.

**Meaning:**

The results of this study suggest that prevention approaches to management of type 2 diabetes may be succeeding in reducing additional cardiovascular risk in patients with heart failure, but there is an urgent need for earlier clinical management of noncardiovascular comorbidities and patient-centered multimorbidity care.

## Teaser

This cohort study investigates changes in cause-specific outcomes and trends associated with type 2 diabetes among individuals with incident heart failure in the United Kingdom from 1998 to 2017.

## Introduction

Type 2 diabetes and heart failure (HF) are 2 of the most prevalent chronic diseases in older people, with numbers projected to rise by 50% over the next 2 decades.^1,2^ Type 2 diabetes is associated with up to a 3-fold increase in the risk of developing HF,^3^ so the conditions frequently coexist. Between 25% and 50% of patients with HF have type 2 diabetes,^4,5^ which is associated with deleterious effects, including increased risk of all-cause death,^6,7,8^ cardiovascular disease (CVD) death,^9,10^ hospitalization for HF,^11^ and rehospitalization.^12,13^

Coronary artery disease (CAD) and hypertension are well-known mediators in the causal pathway between type 2 diabetes and HF.^5^ However, better and earlier primary prevention strategies for cardiovascular health among patients with type 2 diabetes have been associated with declines in major cardiovascular events,^14^ and patients with type 2 diabetes now present more frequently with HF without a prior ischemic event.^15^ Emerging understanding about diabetes-related cardiomyopathy in the absence of CAD has triggered a growing interest in the direct effects of type 2 diabetes on HF incidence and outcomes.^16^ Multiple mechanisms in type 2 diabetes, including hyperglycemia, insulin resistance, lipotoxicity, and microvascular inflammation, can act directly on the myocardium, leading to restrictive or dilated cardiomyopathy.^17^ These direct mechanisms in individuals with type 2 diabetes, alongside the rising prevalence of obesity in this group, means that HF with preserved ejection fraction is becoming an increasingly important phenotype.^18^

The changing phenotype of patients with type 2 diabetes and HF over recent decades is likely to result in a parallel change in risk factors and outcomes for these patients. However, trend data on outcomes and characteristics of patients with type 2 diabetes and HF are scarce and have primarily focused on short-term mortality outcomes in hospital cohorts.^19,20,21,22^ Given the current and projected increase in the health and economic burden of these 2 diseases, insights into these changes are of major importance to provide guidance into new potential therapeutic targets and to develop dynamic and responsive prevention approaches. Our study hypotheses were as follows: (1) in the general HF population, type 2 diabetes is associated with cause-specific short-term and long-term outcomes (ie, hospitalization and mortality) over a 20-year period, and (2) temporal trends in outcomes in HF differ by cause and type 2 diabetes status.

## Methods

### Study Population

We identified all patients with a first diagnosis of HF recorded in their clinical record between January 1, 1998, and July 31, 2017. We used 2 databases, the Clinical Practice Research Datalink (CPRD) and the Hospital Episode Statistics (HES). The CPRD is the largest collection of routinely recorded primary care data globally^13^; it has been validated for epidemiological research^23^ and includes an age-representative and sex-representative sample of the UK general population (accounting for approximately 7% of the population). The HES database includes all admissions to National Health Service hospitals in England. We used 2 further linked databases, the Index of Multiple Deprivation and the Office of National Statistics, which provided measures of socioeconomic status and cause of death, respectively.

Patients were included if they were 30 years or older at the time of HF and were eligible for data linkage (eFigure 1 in the Supplement). Patients in HES were included if they had an *International Statistical Classification of Diseases and Related Health Problems, Tenth Revision *(*ICD*-*10*) diagnostic code for HF in the primary discharge position and had linked CPRD data. To ascertain the HF index date and assess risk factors, all patients required a minimum of 12 months of up-to-standard CPRD data prior to study entry. Up-to-standard is a quality marker indicating that patient data are continuous and complete. When patients had HF codes in both data sets, the earlier code was used as the HF index date. We used an updated, clinically validated HF CPRD code set^24^ and *ICD-10* code set (eTable 1 and eTable 2 in the Supplement).

### Ethical Review

The study protocol was approved by the Independent Scientific Advisory Committee for data access. Ethics approval for use of CPRD data following approval from the Independent Scientific Advisory Committee was granted by a national research ethics committee. While individual patient consent was not required, all data were deidentified, and patients could opt out of data contribution. This study followed the Strengthening the Reporting of Observational Studies in Epidemiology (STROBE) reporting guideline.

### Exposure

We identified patients with HF and diabetes using a detailed set of Read codes (ie, primary care classification in the United Kingdom) and *ICD*-*10* codes (ie, hospital care) applied to clinical records up to and including the HF index date (data available on request). We then applied a detailed algorithm to identify patients with type 2 diabetes,^25^ which has been applied in other CPRD studies^26^ and combines codes with medications, age at diabetes onset, and body mass index (BMI, calculated as weight in kilograms divided by height in meters squared) (eFigure 2 in the Supplement). Patients with type 1 diabetes were excluded. Glucose-lowering medications at baseline were identified from CPRD records in a 4-month window before the HF index date and included metformin, sulphonylureas, thiazolidinediones, incretins, other oral medications, and insulin. Based on previously published work,^7^ patients with both HF and type 2 diabetes were also categorized by medication, as follows: none, oral only, oral plus insulin, and insulin only.

### Baseline Characteristics

We collected information on ischemic heart disease and myocardial infarction as well as other common comorbidities. We used Read and *ICD*-*10* codes in CPRD and HES, respectively, to ascertain comorbidities recorded before and including the HF index date. We also collected information on other risk factors using the most recent measure before study entry, including smoking and alcohol status, BMI, systolic blood pressure, total cholesterol level, hemoglobin level, and estimated glomerular filtration rate. Socioeconomic status was based on the 2010 patient-level Index of Multiple Deprivation score, which was ranked into quintiles (with quintile 1 representing the most affluent group and quintile 5, the most deprived group). Race/ethnicity was categorized into 4 distinct groups, reflecting the most prevalent racial/ethnic groups in the 2011 census in England and Wales, as follows: white, South Asian, black, or other, which included those coded as mixed race/ethnicity, other race/ethnicity, or unknown.

### Hospitalizations and Mortality

All nonelective hospital admissions with at least 1 overnight stay that occurred after but not including the HF index date were included. Admissions during follow-up were counted for each patient and further stratified according to the primary discharge code into HF admissions (*ICD*-*10* chapter 9, HF codes), other cardiovascular admissions (remaining *ICD*-*10* chapter 9 codes), and noncardiovascular admissions (other *ICD*-*10* codes) (eTable 2 in the Supplement). The Office of National Statistics database was used to assign cause of death as cardiovascular when there was an *ICD*-*10* chapter 9 code in the primary position and noncardiovascular for all remaining deaths.

### Statistical Analysis

Data are presented as number and percentage for categorical data, mean and SD for continuous data, and median and interquartile range (IQR) for skewed continuous data. Statistical significance was set at *P* < .05, and all tests were 2-tailed. All analyses were performed in Stata MP version 14.1 (StataCorp).

#### Overall Differences in Outcomes According to Type 2 Diabetes Status

As rates were overdispersed, association of type 2 diabetes with all-cause and cause-specific unplanned hospitalization in patients with HF was estimated using negative binomial models to estimate adjusted incidence rate ratios (IRRs) with 95% CIs. Adjustment included patient factors (ie, age, sex, race/ethnicity, and socioeconomic status), HF factors (ie, place of diagnosis and cardiovascular medications), lifestyle factors (ie, smoking and alcohol status), and clinical factors (ie, comorbidities, BMI, blood pressure, cholesterol level, hemoglobin level, and estimated glomerular filtration rate). For time to mortality outcomes, Royston-Parmar-Lambert flexible parametric survival models were used to estimate age-standardized survival at 1, 3, and 5 years, stratified by diabetes status and adjusted for the same covariates. Age-standardized and calendar year–standardized survival curves for all-cause mortality and cumulative incidence curves for cause-specific mortality were calculated using the stpm2_standsurv and stpm2cif commands.^27^ Adjusted hazard ratios (HRs) with 95% CIs were estimated for all-cause and cause-specific mortality.

To assess the association of glucose-lowering treatment with outcomes, we also stratified the type 2 diabetes group by medication categories for both outcomes, as follows: none, oral only, oral plus insulin, and insulin only (eTable 3 in the Supplement). To account for missing data in the multivariable models, multiple imputations using chained equations were performed using MI Impute in Stata for all variables with missing data (eTable 4 in the Supplement), and results were obtained using Rubin rules to combine 10 imputed data sets.^28^ We performed a sensitivity analysis to assess complete case analysis, removing those with missing values imputed.

#### Outcome Rates and Trends

Absolute rates of hospitalization and mortality were estimated using negative binomial models. Given the higher risk of poor prognosis during the first year after HF diagnosis, outcome rates were calculated separately for the first year (first-year rates) and after the first year (subsequent-year rates). Additionally, owing to the high risk of death in the first month after HF diagnosis, first-year mortality rates were only calculated among survivors of the first month. First-month deaths were presented separately. Using the mean population age and averaged over calendar time, overall rates were estimated for patients with HF with and without type 2 diabetes.

Next, to investigate temporal changes, rates were estimated for two 4-year windows at the start and end of the study, as follows: period 1, 1998 to 2001; and period 2, 2012 to 2015. To assess trends more closely, rates were then estimated for each calendar year of HF diagnosis and examined visually, by plotting graphs of outcome rates by type 2 diabetes status, and analytically, by estimating the *P* value for an interaction term between type 2 diabetes status and HF diagnostic year (as a continuous variable) fitted to the models already containing age. Joinpoint regression was used to estimate the average annual percentage change in outcome rates for those with and without type 2 diabetes and to identify whether rates changed in any specific calendar year. Risk factor trends were estimated at the mean population age for each calendar year of diagnosis, using logit for binary risk factors or linear models for continuous risk factors.

## Results

### Study Population

There were 87 709 patients with incident HF during the 20-year period, including 43 173 women (49.2%) and 78 211 white individuals (89.2%); the median (IQR) follow-up was 2.36 (0.46-5.67) years. Preexisting type 2 diabetes was present in 20 858 patients (23.8%) with HF. Comparing patients with HF with type 2 diabetes vs 66 851 patients (76.2%) without diabetes, patients with type 2 diabetes were younger at HF diagnosis (median [IQR] age, 78.0 [70.0-84.0] vs 80.0 [72.0-86.0] years), were more likely to be diagnosed in the hospital setting (10 569 [50.7%] vs 25 525 [38.2%]), were more likely to be men (11 453 [54.9%] vs 33 083 [49.5%]), were more likely to belong to the most deprived quintile (4086 [19.6%] vs 10 659 [16.0%]), and had more comorbidities (mean [SD] comorbidities, 5.4 [1.9] vs 3.7 [1.9]) (eTable 5 in the Supplement). In terms of cardiovascular risk factors, patients with type 2 diabetes were more likely to be prescribed cardiovascular medications at the time of HF diagnosis (eg, 7287 [34.9%] vs 19 134 [28.6%] prescribed β-blocker; 9358 [44.9%] vs 23 375 [35.0%] prescribed angiotensin-converting enzyme inhibitor), to have lower systolic blood pressure (mean [SD], 136.9 [20.9] mm Hg vs 138.2 [21.9] mm Hg) and cholesterol levels (median [IQR], 162.2 [135.1-193.1] mg/dL vs 181.5 [154.4-216.2] mg/dL [to convert to millimoles per liter, multiply by 0.0259]), and less likely to be active smokers (3822 [18.7%] vs 13 382 [21.5%]). Conversely, they had a higher BMI (mean [IQR] 29.0 [25.3-33.6] vs 26.2 [23.1-29.8]) and lower hemoglobin levels (mean [SD], 12.7 [1.9] g/dL vs 13.1 [1.9] g/dL [to convert to grams per liter, multiply by 10.0]) (*P* < .001 for all comparisons).

### Overall Differences in Hospitalization Rates

Following adjustment for all covariates, the group with type 2 diabetes was associated with an approximately 25% higher rate of unplanned hospital admission compared with the group without diabetes (IRR for CVD hospitalization, 1.24; 95% CI, 1.19-1.30; IRR for non-CVD hospitalization, 1.26; 95% CI, 1.22-1.30) (Table 1). When the type 2 diabetes group was stratified by glucose-lowering drug therapy and compared with the group without diabetes, the highest risk was associated with patients prescribed insulin only (IRR for CVD hospitalization, 1.47; 95% CI, 1.34-1.60; IRR for non-CVD hospitalization, 1.68; 95% CI, 1.57-1.80). When stratified by calendar year in the sensitivity analysis, the adjusted risk associated with type 2 diabetes reduced significantly for CVD hospitalization from an IRR of 1.36 (95% CI, 1.22-1.52) in period 1 to an IRR of 1.13 (95% CI, 1.02-1.24) in period 2. This decrease was not observed for non-CVD hospitalizations (IRR for period 1, 1.23; 95% CI, 1.14-1.32; IRR for period 2, 1.21; 95% CI, 1.13-1.30) (Table 1).

**Table 1.** Associations Between Type 2 Diabetes Status and Hospital Admissions in Incident HF

Group	Total Hospital Admissions, No.	Follow-up		Incidence Rate Ratio (95% CI)					
				All		CVD		Non-CVD	
		Total PY	Median (IQR), y	Unadjusted	Adjusted^a^	Unadjusted	Adjusted^a^	Unadjusted	Adjusted^a^
HF without diabetes	161 109	261 602	2.5 (0.5-6.0)	1 [Reference]	1 [Reference]	1 [Reference]	1 [Reference]	1 [Reference]	1 [Reference]
HF with type 2 diabetes	64 386	66 141	2.0 (0.4-4.8)	1.46 (1.43-1.49)	1.25 (1.21-1.29)	1.60 (1.55-1.66)	1.24 (1.19-1.30)	1.44 (1.40-1.47)	1.26 (1.22-1.30)
1998-2002	50 654	84 495	3.0 (0.5-7.5)	1.47 (1.39-1.55)	1.27 (1.18-1.36)	1.78 (1.64-1.93)	1.36 (1.22-1.52)	1.35 (1.27-1.43)	1.23 (1.14-1.32)
2012-2015	33 136	40 652	2.2 (0.7-3.6)	1.41 (1.34-1.48)	1.18 (1.11-1.26)	1.45 (1.35-1.56)	1.13 (1.02-1.24)	1.42 (1.35-1.50)	1.21 (1.13-1.30)
No medication	23 664	22 507	1.5 (0.3-3.8)	1.52 (1.47-1.57)	1.21 (1.16-1.25)	1.60 (1.53-1.68)	1.20 (1.13-1.27)	1.53 (1.48-1.59)	1.22 (1.17-1.26)
Oral medication only^b^	26 519	30 752	2.4 (0.4-5.4)	1.31 (1.27-1.35)	1.20 (1.16-1.24)	1.49 (1.43-1.56)	1.22 (1.15-1.29)	1.27 (1.22-1.31)	1.19 (1.15-1.24)
Oral medication plus insulin^b^	7086	6926	2.9 (0.9-5.6)	1.49 (1.40-1.58)	1.45 (1.36-1.54)	1.75 (1.61-1.91)	1.39 (1.27-1.53)	1.45 (1.35-1.55)	1.47 (1.37-1.57)
Insulin only	7117	5955	2.1 (0.5-4.9)	1.87 (1.75-1.99)	1.57 (1.48-1.68)	1.98 (1.80-2.17)	1.47 (1.34-1.60)	1.89 (1.76-2.02)	1.68 (1.57-1.80)

Abbreviations: CVD, cardiovascular disease; HF, heart failure; IQR, interquartile range; PY, person-years.

^a^ Adjusted for age, sex, socioeconomic status, race/ethnicity, place of diagnosis, calendar year, prescriptions (ie, β-blocker, angiotensin-converting enzyme inhibitor, angiotensin receptor blocker, aldosterone antagonist, aspirin, or diuretic), number of comorbidities, ischemic heart disease, myocardial infarction, atrial fibrillation, hypertension, diabetes, stroke, anemia, obesity, chronic kidney disease, chronic obstructive pulmonary disease, asthma, depression, osteoarthritis, cancer, dementia, smoking status, alcohol use, body mass index, systolic blood pressure, cholesterol level, hemoglobin level, and estimated glomerular filtration rate.

^b^ Oral medication refers to oral glucose-lowering drugs, including metformin, sulphonylureas, thiazolidinediones, incretins, and others.

### Hospitalization Rates

#### First-Year Rates

**Figure 1.**
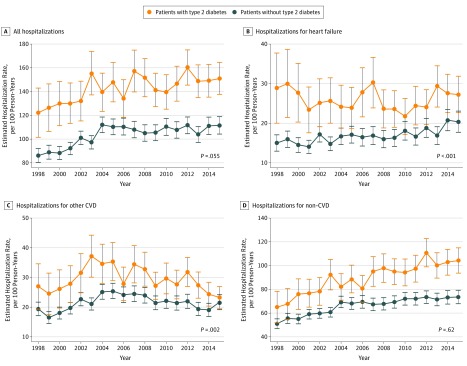
Trends in Estimated 1-Year Rates of Cause-Specific Hospitalization Estimated admission rates at mean population age (78 years) per 100 person-years between 1998 and 2015. Follow-up was until death or study end. Error bars indicate 95% CIs. *P* values are for the test of difference in trends. CVD indicates cardiovascular disease.

Age-adjusted hospitalization rates were significantly higher for patients with HF and type 2 diabetes (145.9 per 100 person-years [PYs]; 95% CI, 142.1 to 149.7 per 100 PYs) than without (103.8 per 100 PYs; 95% CI, 102.2 to 105.4 per 100 PYs) (Table 2 and Figure 1A). First-year rates increased similarly for both groups between period 1 and period 2 (HF with type 2 diabetes, 133.3 [95% CI, 102.2 to 105.4] per 100 PYs vs 152.5 [95% CI, 145.5 to 159.5] per 100 PYs; HF with no diabetes, 89.7 [95% CI, 86.8 to 92.5] per 100 PYs vs 110.0 [95% CI, 106.5 to 113.6] per 100 PYs; *P* for difference in trends = 0.06) (Table 2 and Figure 1A). However, there were noticeable trend differences by cause of admission. While patients without diabetes experienced a steady and consistent 1.7% (95% CI, 1.1% to 2.3%) annual increase in hospital admissions for HF during their first year after diagnosis, the group with type 2 diabetes experienced a −2.2% (95% CI, −3.9% to −0.5%) annual reduction until 2010 (*P* for difference in trends < .001) (Table 2 and Figure 1B). Other CVD admissions increased for both groups until 2005 (HF without diabetes, 4.9%; 95% CI, 2.1% to 7.8%; HF with type 2 diabetes, 5.4%; 95% CI, 1.6% to 9.4%) and then decreased (HF without diabetes, −2.3%; 95% CI, −3.8% to −0.8%; HF with type 2 diabetes, −3.1%; 95% CI, −4.5% to −1.6%), with an overall small reduction in predicted rate per 100 PYs among patients with type 2 diabetes (period 1, 28.1; 95% CI, 24.8 to 31.3; period 2, 26.4; 95% CI, 24.3 to 28.5) and an increase among those without diabetes (period 1, 18.9; 95% CI, 17.8 to 19.9; period 2, 20.5; 95% CI, 19.3 to 21.7) (*P* for difference in trends = .002) (Table 2 and Figure 1C). Non-CVD admissions increased for both groups (HF without diabetes, 4.5%; 95% CI, 3.4% to 5.6%; HF with type 2 diabetes, 1.0%; 95% CI, 0.6% to 1.4%) until 2004. After 2004, rates continued to increase, at a lower rate for patients without diabetes (1.1%; 95% CI, 0.8% to 1.4%) and at a notably (but not statistically significant) higher rate among those with type 2 diabetes (2.3%; 95% CI, 0.9% to 3.6%) (Table 2 and Figure 1D).

**Table 2.** Estimated Rates of Admissions by Diabetes Status and Calendar Year

Group	Estimated Rate per 100 Person-Years (95% CI)			*P* Value for Interaction^a^	Annual Mean Change in Rates, % (95% CI)^b^	Year of Change	New Annual Change in Rates, % (95% CI)^b^
	Overall	1998-2001	2012-2015				
**All Hospitalizations**							
First year after heart failure diagnosis							
HF without diabetes	103.8 (102.2 to 105.4)	89.7 (86.8 to 92.5)	110.0 (106.5 to 113.6)	.06	4.1 (2.4 to 5.9)	2004	0.2 (−0.4 to 0.8)
HF with type 2 diabetes	145.9 (142.1 to 149.7)	133.3 (124.0 to 142.7)	152.5 (145.5 to 159.5)		0.9 (0.1 to 1.7)	NA	NA
Subsequent years							
HF without diabetes	76.1 (75.2 to 77.1)	75.3 (73.4 to 77.2)	71.5 (69.4 to 73.7)	.08	−0.3 (−0.9 to 0.3)	NA	NA
HF with type 2 diabetes	123.5 (120.7 to 126.3)	117.6 (110.7 to 124.5)	108.8 (104.1 to 113.4)		−0.3 (−2.1 to 1.6)	NA	NA
**HF Hospitalizations**							
First year after heart failure diagnosis							
HF without diabetes	16.9 (16.4 to 17.4)	14.9 (14.0 to 15.9)	19.4 (18.1 to 20.6)	<.001	1.7 (1.1 to 2.3)	NA	NA
HF with type 2 diabetes	25.9 (24.6 to 27.2)	28.7 (25.0 to 32.5)	27.5 (25.0 to 29.9)		−2.2 (−3.9 to −0.5)	2010	4.6 (−2.2 to 11.8)
Subsequent years							
HF without diabetes	7.5 (7.3 to 7.8)	7.4 (7.0 to 7.9)	7.6 (7.0 to 8.2)	.12	−0.0 (−0.9 to 0.8)	NA	NA
HF with type 2 diabetes	15.3 (14.5 to 16.1)	16.7 (14.5 to 18.8)	14.6 (13.1 to 16.0)		−0.8 (−1.8 to 0.3)	NA	NA
**Other-CVD Hospitalizations**							
First year after heart failure diagnosis							
HF without diabetes	21.7 (21.1 to 22.2)	18.9 (17.8 to 19.9)	20.5 (19.3 to 21.7)	.002	4.9 (2.1 to 7.8)	2005	−2.3 (−3.8 to −0.8)
HF with type 2 diabetes	29.4 (28.1 to 30.7)	28.1 (24.8 to 31.3)	26.4 (24.3 to 28.5)		5.4 (1.6 to 9.4)	2004	−3.1 (−4.5 to −1.6)
Subsequent years							
HF without diabetes	11.9 (11.6 to 12.2)	12.9 (12.4 to 13.4)	10.1 (9.5 to 10.7)	.046	−1.5 (−2.2 to −0.8)	NA	NA
HF with type 2 diabetes	19.6 (18.8 to 20.3)	21.5 (19.5 to 23.5)	15.1 (13.9 to 16.3)		1.4 (−0.0 to 2.9)	2007	−6.6 (−8.6 to −4.6)
**Non-CVD Hospitalizations**							
First year after heart failure diagnosis							
HF without diabetes	66.5 (65.3 to 67.7)	55.7 (53.7 to 57.8)	73.7 (70.9 to 76.4)	.62	4.5 (3.4 to 5.6)	2004	1.1 (0.8 to 1.4)
HF with type 2 diabetes	93.0 (90.2 to 95.8)	75.2 (68.9 to 81.5)	104.6 (99.1 to 110.1)		1.0 (0.6 to 1.4)	2004	2.3 (0.9 to 3.6)
Subsequent years							
HF without diabetes	57.2 (56.4 to 58.0)	54.7 (53.2 to 56.2)	55.5 (53.7 to 57.4)	.37	0.2 (−0.4 to 0.7)	NA	NA
HF with type 2 diabetes	91.2 (89.0 to 93.5)	80.4 (75.3 to 85.5)	83.5 (79.7 to 87.4)		0.6 (−0.8 to 2.0)	NA	NA

Abbreviations: CVD, cardiovascular disease; HF, heart failure; NA, not applicable.

^a^ *P* value for the difference in trend lines between groups. Estimated by fitting an interaction term between calendar year as a continuous variable and type 2 diabetes status in the negative binomial models also containing age.

^b^ Mean annual percentage change in rates (per 100 person-years) for each increasing year of diagnosis. Any significant change in the trend line for rates was estimated using joinpoint regression. When present, rates are reported before and after the year of change.

#### Subsequent-Year Rates

With the exception of other CVD hospitalizations, subsequent-year rates remained stable over time (Table 2). Rates for other CVD admissions decreased at a significantly greater rate among patients with type 2 diabetes after 2007 (−6.6%; 95% CI, −8.6% to −4.6%) than among patients without diabetes (−1.5%; 95% CI, −2.2% to −0.8%) (*P* for difference in trends = .046).

### Mortality Rates

#### Overall Differences in Mortality

In patients with HF, type 2 diabetes was associated with an increased risk of all-cause death (adjusted HR, 1.14; 95% CI, 1.11-1.18) and CVD death (adjusted HR, 1.06; 95% CI, 1.02-1.10) and was highest for non-CVD death (adjusted HR, 1.24; 95% CI, 1.19-1.29). When stratified by calendar year, type 2 diabetes was associated with increased risk of non-CVD death (adjusted HR, 1.14; 95% CI, 1.04-1.25) but not with CVD death (adjusted HR, 1.00; 95% CI, 0.90-1.09) in period 2 (Table 3).

**Table 3.** Associations Between Type 2 Diabetes Status and Death in Incident HF

Group	Total Deaths, No. (%)	Survival Time, Median (IQR), y	Age at Death, Median (IQR), y	Hazard Ratios (95% CI)					
				All		CVD		Non-CVD	
				Unadjusted	Adjusted^a^	Unadjusted	Adjusted^a^	Unadjusted	Adjusted^a^
HF without diabetes	49 604 (74.2)	3.3 (3.2-3.3)	85 (79-90)	1 [Reference]	1 [Reference]	1 [Reference]	1 [Reference]	1 [Reference]	1 [Reference]
HF with type 2 diabetes	14 884 (71.4)	2.9 (2.8-2.9)	82 (75-87)	1.12 (1.10-1.14)	1.14 (1.11-1.18)	1.18 (1.15-1.21)	1.06 (1.02-1.10)	1.06 (1.03-1.09)	1.24 (1.19-1.29)
1998-2002	16 259 (93.8)	3.0 (2.9-3.1)	84 (77-89)	1.17 (1.12-1.22)	1.25 (1.18-1.33)	1.32 (1.24-1.41)	1.22 (1.11-1.33)	1.08 (1.02-1.14)	1.28 (1.18-1.39)
2012-2015	9023 (50.9)	3.4 (3.3-3.5)	86 (79-90)	1.11 (1.06-1.16)	1.07 (1.10-1.13)	1.11 (1.05-1.18)	1.00 (0.90-1.09)	1.11 (1.04-1.18)	1.14 (1.04-1.25)
No medication	5615 (66.0)	2.5 (2.4-2.6)	84 (77-89)	1.17 (1.14-1.21)	1.09 (1.05-1.13)	1.19 (1.15-1.24)	0.99 (0.95-1.04)	1.15 (1.11-1.20)	1.21 (1.15-1.27)
Oral medication only^b^	6535 (74.8)	3.1 (3.0-3.2)	82 (76-87)	1.08 (1.06-1.11)	1.15 (1.11-1.18)	1.17 (1,12-1.21)	1.09 (1.04-1.14)	1.01 (0.97-1.04)	1.21 (1.15-1.27)
Oral medication plus insulin^b^	1261 (68.6)	4.0 (3.7-4.3)	77 (70-83)	0.94 (0.89-0.99)	1.28 (1.21-1.36)	1.05 (0.98-1.14)	1.22 (1.12-1.32)	0.82 (0.75-0.89)	1.35 (1.23-1.48)
Insulin only	1473 (83.3)	2.4 (2.2-2.6)	79 (72-85)	1.25 (1.19-1.32)	1.37 (1.30-1.46)	1.30 (1.21-1.39)	1.20 (1.11-1.29)	1.20 (1.11-1.29)	1.59 (1.47-1.73)

Abbreviations: CVD, cardiovascular disease; HF, heart failure; IQR, interquartile range.

^a^ Adjusted for age, sex, socioeconomic status, race/ethnicity, place of diagnosis, calendar year, prescriptions (ie, β-blocker, angiotensin-converting enzyme inhibitor, angiotensin receptor blocker, aldosterone antagonist, aspirin, or diuretic), number of comorbidities, ischemic heart disease, myocardial infarction, atrial fibrillation, hypertension, diabetes, stroke, anemia, obesity, chronic kidney disease, chronic obstructive pulmonary disease, asthma, depression, osteoarthritis, cancer, dementia, smoking status, alcohol use, body mass index, systolic blood pressure, cholesterol level, hemoglobin level, and estimated glomerular filtration rate.

^b^ Oral medication refers to oral glucose-lowering drugs, including metformin, sulphonylureas, thiazolidinediones, incretins, and others.

#### Trends in Mortality Rates

**Figure 2.**
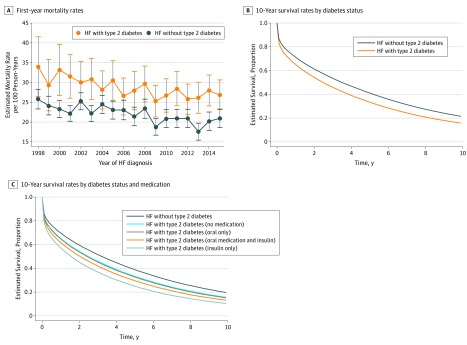
Trends in Estimated Mortality Rates and Survival by Diabetes Status A, Estimated mortality rates at mean population age (78 years) per 100 person-years between 1998 and 2015. Error bars indicate 95% CIs. Rates were calculated among survivors of the first month following HF diagnosis. *P* for difference in trend < .001. HF indicates heart failure.

During the follow-up period, 14 884 patients with type 2 diabetes (71.4%) died (median [IQR] survival, 2.9 [2.8 to 2.9] years) and 49 604 patients without diabetes (74.2%) died (median [IQR] survival, 3.3 [3.2 to 3.3] years) (Table 3). First-year, age-standardized mortality rates were significantly higher in patients with type 2 diabetes (28.5 per 100 PYs; 95% CI, 27.5 to 30.0 per 100 PYs) compared with those without diabetes (22.4 per 100 PYs; 95% CI, 21.7 to 23.1 per 100 PYs), but the gap narrowed over time, owing to more quickly decreasing rates in the type 2 diabetes group (−1.4% [95% CI, −1.8% to −0.9%] vs −0.7% [95% CI, −1.2% to −0.2%] per year; *P* for difference in trends < .001) (Figure 2A; eTable 6 in the Supplement). This trend was similar for subsequent-year mortality rates over a longer follow-up period. Age-standardized and calendar year–standardized survival were lower in the type 2 diabetes group (Figure 2B) and lowest in the type 2 diabetes group prescribed insulin only (Figure 2C). In those without diabetes, 1-year, 3-year, and 5-year age-standardized mortality risk was 29.2% (95% CI, 29.0% to 29.5%), 46.7% (46.3% to 47.0%), and 59.2% (95% CI, 58.9% to 59.6%), respectively; these rates were higher among patients with type 2 diabetes, at 35.6% (95% CI, 35.1% to 36.1%), 54.6% (95% CI, 54.0% to 55.1%), and 67.2% (95% CI, 66.7% to 67.7%), respectively (eTable 7 in the Supplement). Risk reduced by approximately 4% to 5% for both groups between 1998-2001 and 2012-2015 (for 1-year risk among patients without diabetes, 30.9% [95% CI, 30.5% to 31.4%] vs 27.0% [26.5% to 25.74%]; with type 2 diabetes, 37.5% [95% CI, 36.8% to 38.1%] vs 33.0% [95% CI, 32.4% to 33.6%]) (eTable 7 in the Supplement). For both groups, age-standardized and calendar year–standardized risk of cardiovascular death was higher than risk of non-CVD death, but the 2 causes converged, and non-CVD death became the primary cause of death from approximately 4 years of follow-up (eFigure 3 in the Supplement). In complete case analysis, associations of type 2 diabetes with hospitalization and mortality rates were similar (eTable 8 in the Supplement).

#### Trends in Risk Factors

Between 1998 and 2017, age at HF onset for those with type 2 diabetes and without diabetes converged, owing to more quickly increasing age among patients with type 2 diabetes over time (*P* for difference in trends < .001) (eFigure 4 in the Supplement). Smoking prevalence reduced similarly between groups (*P* for difference in trends = .65). While systolic blood pressure reduced at a slightly slower rate among patients with type 2 diabetes, the most noticeable trend differences were for BMI, hemoglobin level, and cholesterol level. Patients with type 2 diabetes had a significantly steeper increase in BMI (*P* for difference in trends < .001) and decrease in hemoglobin level (*P* for difference in trends = .046). However, cholesterol levels remained significantly lower in patients with type 2 diabetes and reduced at a much faster rate before 2008 (*P* for difference in trends < .001). For both groups, the number of comorbidities increased, while the prevalence of ischemic heart disease decreased after 2008 (*P *for difference in trends = .67) (eFigure 5 in the Supplement). Patients with type 2 diabetes had faster increasing rates of hypertension and iron deficiency anemia and more slowly decreasing rates of chronic kidney disease (*P* for difference in trends < .001). Obesity increased similarly for both groups (*P *for difference in trends = .96) (eFigure 5 in the Supplement).

## Discussion

In this national population-based study, people with type 2 diabetes developed HF at a younger age and had significantly worse outcomes than those without diabetes, with an approximately 25% higher risk of all-cause unplanned hospitalization and an approximately 15% higher risk of all-cause death. The higher risk associated with type 2 diabetes persisted over 2 decades, but there were significant differences in cause-specific trends in patients with type 2 diabetes and those without diabetes. Type 2 diabetes was associated with higher risk of CVD outcomes, but the difference between the groups converged over time; by period 2 (ie, 2012-2015), the relative risk of CVD hospitalization had halved among patients with type 2 diabetes, while there was no evidence of an increased risk of CVD mortality. Type 2 diabetes was also associated with higher risk of non-CVD outcomes, but in contrast to CVD findings, this risk persisted over time, and differences in non-CVD hospitalization rates between patients with type 2 diabetes and those without increased. These outcome patterns were aligned with differences in observed risk factor trends between the 2 groups. While the prevalence of ischemic heart disease had begun to decrease for both groups in the last decade, patients with type 2 diabetes at HF onset were more likely to be prescribed cardiovascular medications and had a better cardiovascular risk profile, with lower systolic blood pressure, cholesterol levels, and prevalence of smoking. Conversely, patients with type 2 diabetes had more comorbidities, including a faster increase in BMI, a higher prevalence of hypertension and iron deficiency anemia, and more slowly reducing prevalence of renal disease.

Four hospital-based studies from Europe^19,20^ and the United States^21,22^ have investigated trends in the characteristics and outcomes of patients with HF and type 2 diabetes. All studies were hospital-based and, with 1 exception,^20^ focused on all-cause, in-hospital mortality or hospital readmissions. Collectively, these studies showed type 2 diabetes to be associated with decreased in-hospital mortality, with similar group trends over time. A study in Spain^19^ reported increasing all-cause readmission trends in those with and without type 2 diabetes. In a study with longer follow-up (ie, 10 years) after intensive care unit admission,^20^ type 2 diabetes was associated with a 17% increase risk of all-cause mortality, with small reductions in mortality rates over time, similar to the findings of our study in a general HF population. Previous, nontrend outcome studies in type 2 diabetes and HF have focused on all-cause and CVD outcomes, showing type 2 diabetes to be associated with increased risk of all-cause hospitalizations,^7,13^ HF hospitalizations,^29,30^ and all-cause and CVD death.^9,30^ Few data on non-CVD outcomes exist; the Emerging Risk Factor Collaboration^31^ recently found type 2 diabetes to be associated with several non-CVD causes of death, including cancer and respiratory, renal, and liver disorders.

Our study confirmed these findings but also added substantial distinct information. First, we used a general population incident cohort of patients with HF, diagnosed in the community or in hospital, with up to 20 years of follow-up (median [IQR], 2.4 [0.5-5.7] years). Second, we used a range of cause-specific outcomes to perform detailed trend analyses over 2 decades. We reported that, while trends in all-cause unplanned hospitalizations and mortality in patients with HF with and without type 2 diabetes were similar, there were opposing trends in cause-specific outcomes between groups. While HF-specific admissions increased in patients without diabetes, admissions decreased in those with type 2 diabetes, who also experienced a steeper increase in non-CVD admissions. Furthermore, in recent years, there was no longer evidence of an increased risk of CVD death associated with type 2 diabetes, but the risk of non-CVD death was persistently higher. This information is crucial to inform the development of dynamic and responsive prevention approaches.

Current type 2 diabetes guidelines focus on management of glucose and other risk factors to reduce the risk of cardiovascular disease.^32^ However, while early cardiovascular prevention approaches have succeeded in reducing cardiovascular outcomes,^14^ the prevalence of individuals with HF and type 2 diabetes is increasing.^33^ However, until recently (with the exception of the DECLARE-TIMI 58 study^34^), HF has been neglected as a primary outcome in diabetes therapeutic trials.^35,36^ Furthermore, the current findings show the significant and persistent risk posed by type 2 diabetes in individuals with HF, adding to the well-known atherothrombotic complications of diabetes.^37^ Type 2 diabetes–related cardiomyopathy is more prevalent in HF with preserved ejection fraction, now the most common form of HF,^21^ and this may explain the increasing trend in non-CVD admissions and persisting non-CVD mortality risk observed in this group.^38^ Our findings on cause-specific trends indicated that primary and secondary prevention approaches in type 2 diabetes management may be succeeding in reducing additional cardiovascular risk in the population with HF while highlighting the importance of earlier management of noncardiovascular comorbidities, including chronic kidney disease, anemia, and obesity. In this endeavor, recent successes of sodium glucose cotransporter 2 inhibitor^39,40^ and glucagon-like peptide-1 receptor agonist^41,42^ trials in reducing HF admissions in patients with type 2 diabetes show early promise. Initial trials suggest that their effects may be unrelated to glycemic control^43,44^ or the presence of ischemic heart disease,^45^ and a protective effect was observed in patients without type 2 diabetes who experienced HF.^46^ Potential mechanisms are associated with the promotion of diuresis and natriuresis^47^ having salutary hemodynamic and renal effects^48^ and concomitant weight loss.^49^ While trials are ongoing, use of these drugs among patients with type 2 diabetes with established atherosclerotic CVD or with chronic kidney disease has been recently recommended.^50^ While other CVD hospitalizations decreased, the increasing HF hospital admissions in the group without diabetes was surprising. This finding likely represents the increasingly older and multimorbid HF population as well as the increasing number of patients diagnosed first with HF in hospital,^51^ potentially indicating more severe HF at the time of onset and the potential importance of implementing type 2 diabetes cardiovascular risk prevention approaches to other high-risk multimorbid groups earlier on.

### Strengths and Limitations

To our knowledge, this study is the first to report trends in characteristics and cause-specific outcomes in the general population with incident HF and type 2 diabetes. We used linked, nationally representative databases to ascertain the incidence date of HF and observed patients for up to 20 years. However, there were some limitations. This is an observational study using routine data collection, which can be subject to misclassification and changes to coding practices. However, accuracy of clinical recording and diagnoses within the CPRD have been found to be valid for a range of morbidities,^23^ and we also used clinically validated code sets with high precision,^17^ a detailed type 2 diabetes algorithm,^25^ and combined general practice and hospital codes for HF, comorbidities, and cause-specific outcomes. Diagnosis of HF in the general population is clinically defined, and not all patients will undergo echocardiography. While this might lead to some misclassification, HF diagnosis has been enhanced by the introduction of echocardiography as part of performance incentives^52^ and national HF audits. Echocardiography data were not available, so we were not able to differentiate between different HF phenotypes, which may be distinct in terms of outcome trends associated with type 2 diabetes. Also, reducing HF severity among patients with type 2 diabetes, who received better cardiovascular treatment, provides a potential explanation for the diverging trends in HF-specific admissions, but we could not adjust for HF severity. To account for severity, a range of comorbidities and medications were included in our analyses, but further investigation into both hypotheses is required.

## Conclusions

The results of this study suggest that prevention approaches to management of type 2 diabetes may be succeeding in reducing additional cardiovascular risk in patients with type 2 diabetes and heart failure. However, type 2 diabetes in patients with HF in the general population poses a major and persistent public health challenge. The distinct cause-specific outcome trends we identified in this high-risk group promote the use of early cardiovascular risk prevention approaches for other groups with high cardiovascular risk and indicate an urgent need for earlier comorbidity management and patient-centered multimorbidity care in type 2 diabetes to reduce its increasing burden.

## Notes

Supplement. **eFigure 1. **Flow Chart of the Patient Selection Process **eFigure 2. **Algorithm for Categorizing People With Type 1 and Type 2 Diabetes **eFigure 3.** Cumulative Incidence by Cause of Death **eFigure 4. **Trends in Risk Factors **eFigure 5. **Trends in Comorbidities at HF Diagnosis by Diabetes Status **eTable 1. **Heart Failure Selection CPRD Code Set **eTable 2. **Heart Failure Selection *ICD-10* Code Set **eTable 3. **Glucose-Lowering Medications **eTable 4. **Missing Data **eTable 5.** Patient Characteristics at Diagnosis of Heart Failure by Diabetes Status **eTable 6. **Predicted Rates of Death During the First and Subsequent Years Following HF Diagnosis, by Type 2 Diabetes Status and Calendar Year **eTable 7. **Age-Standardized Predicted Mortality Risk by Calendar Time **eTable 8. **Associations With Hospital Admission and Death in Complete Case Analysis
