# Supplementary material for: Trends in Cause-Specific Outcomes Among Individuals With Type 2 Diabetes and Heart Failure in the United Kingdom, 1998-2017
Source: JAMA Netw Open. 2019 Dec 2;2(12):e1916447. doi: 10.1001/jamanetworkopen.2019.16447 (PMC6902744; doi:10.1001/jamanetworkopen.2019.16447)
Supplement: Supplement. — eFigure 1. Flow Chart of the Patient Selection Process eFigure 2. Algorithm for Categorizing People With Type 1 and Type 2 Diabetes eFigure 3. Cumulative Incidence by Cause of Death eFigure 4. Trends in Risk Factors eFigure 5. Trends in Comorbidities at HF Diagnosis by Diabetes Status eTable 1. Heart Failure Selection CPRD Code Set eTable 2. Heart Failure Selection ICD-10 Code Set eTable 3. Glucose-Lowering Medications eTable 4. Missing Data eTable 5. Patient Characteristics at Diagnosis of Heart Failure by Diabetes Status eTable 6. Predicted Rates of Death During the First and Subsequent Years Following HF Diagnosis, by Type 2 Diabetes Status and Calendar Year eTable 7. Age-Standardized Predicted Mortality Risk by Calendar Time eTable 8. Associations With Hospital Admission and Death in Complete Case Analysis [file jamanetwopen-2-e1916447-s001.pdf]

Supplementary Online Content

Lawson CA, Zaccardi F, McCann GP, Davies MJ, Kadam UT, Khunti K. Trends in cause-specific outcomes among individuals with type 2 diabetes and heart failure in the United Kingdom, 1998-2017. *JAMA Netw Open*. 2019;2(12):e1916447. doi:10.1001/jamanetworkopen.2019.16447

eFigure 1. Flow Chart of the Patient Selection Process

eFigure 2. Algorithm for Categorizing People With Type 1 and Type 2 Diabetes

eFigure 3. Cumulative Incidence by Cause of Death

eFigure 4. Trends in Risk Factors

eFigure 5. Trends in Comorbidities at HF Diagnosis by Diabetes Status

eTable 1. Heart Failure Selection CPRD Code Set

eTable 2. Heart Failure Selection *ICD-10* Code Set

eTable 3. Glucose-Lowering Medications

eTable 4. Missing Data

eTable 5. Patient Characteristics at Diagnosis of Heart Failure by Diabetes Status

eTable 6. Predicted Rates of Death During the First and Subsequent Years Following HF Diagnosis, by Type 2 Diabetes Status and Calendar Year

eTable 7. Age-Standardized Predicted Mortality Risk by Calendar Time

eTable 8. Associations With Hospital Admission and Death in Complete Case Analysis

This supplementary material has been provided by the authors to give readers additional information about their work.

© 2019 Lawson CA et al. *JAMA Network Open.*

eFigure 1. Flow Chart of the Patient Selection Process

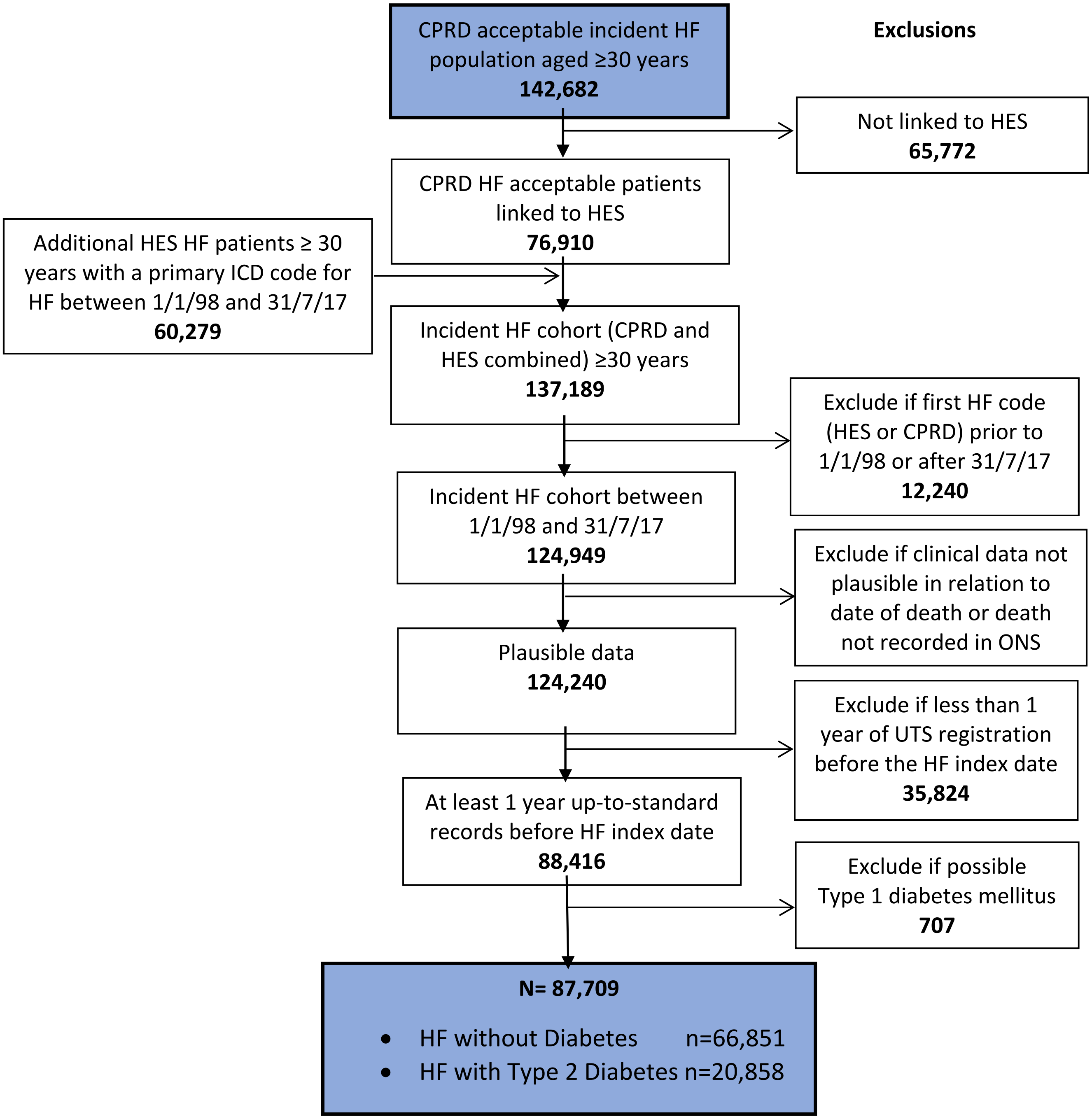

HES: Hospital Episode Statistics; **HF**: Heart Failure; **UTS:** Up to standard.

© 2019 Lawson CA et al. *JAMA Network Open.*

eFigure 2. Algorithm for Categorizing People With Type 1 and Type 2 Diabetes

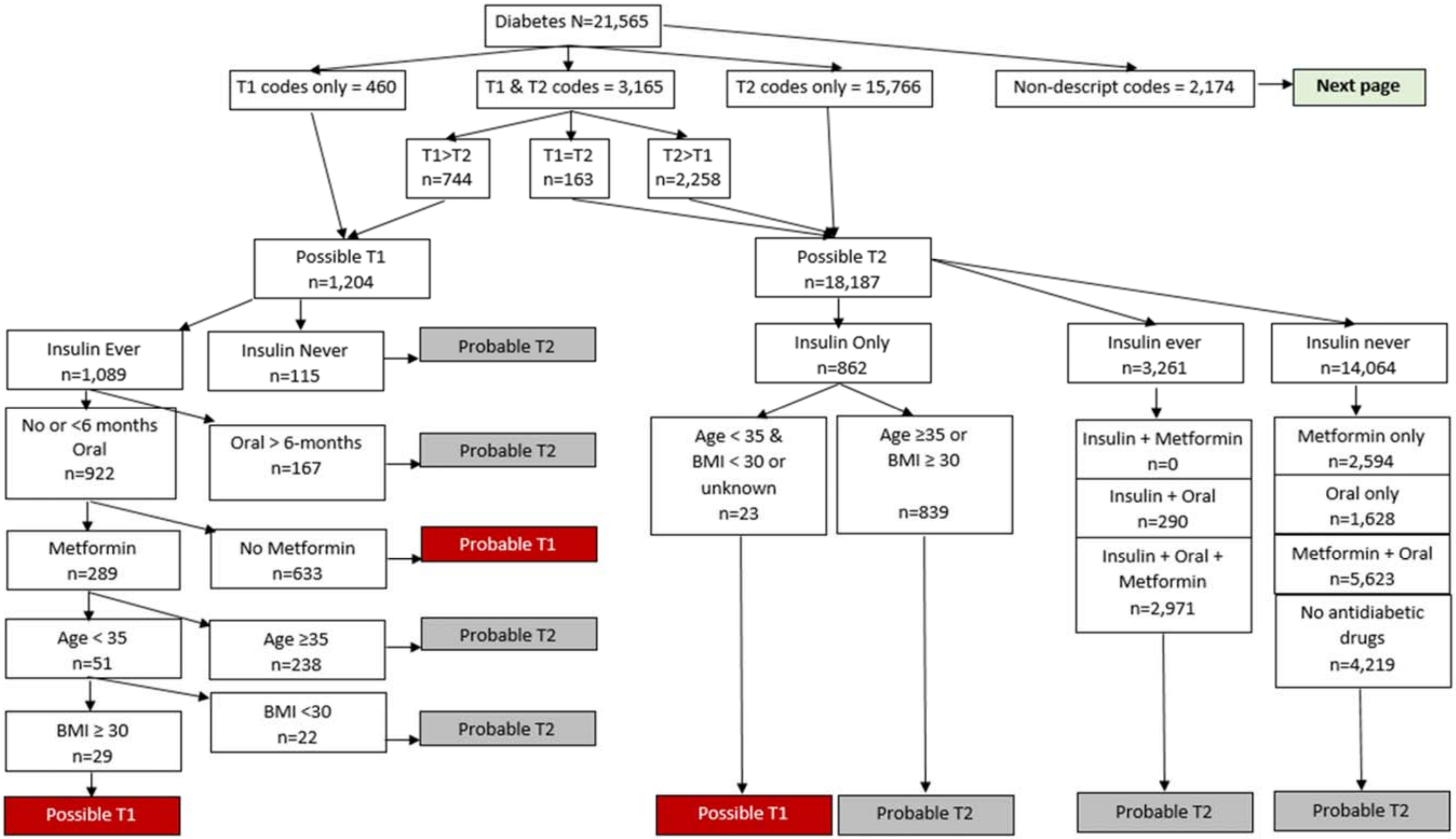

© 2019 Lawson CA et al. *JAMA Network Open.*

eFigure 2. Algorithm for Categorizing People With Type 1 and Type 2 Diabetes (cont.)

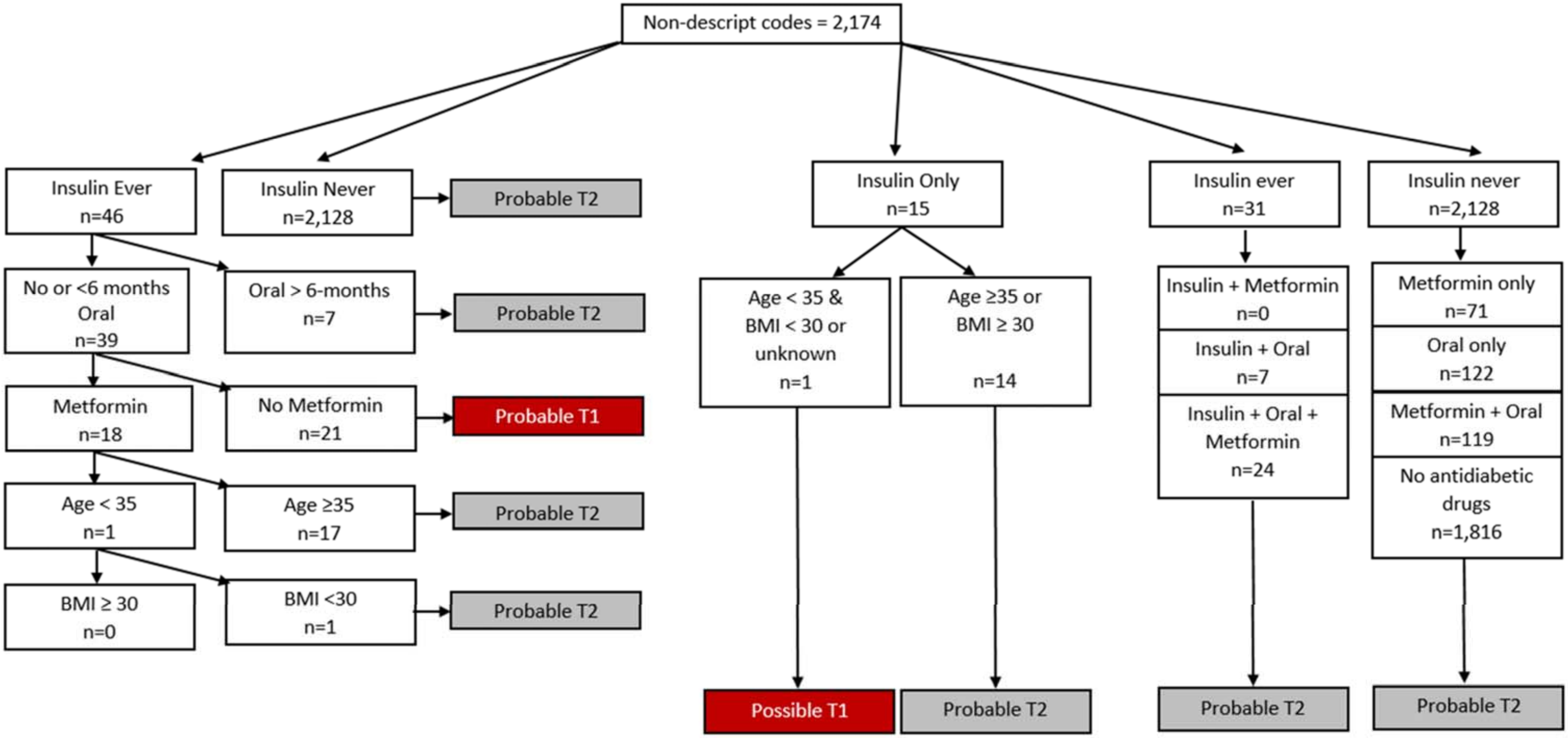

Algorithm for categorising Type 1 and Type 2 Diabetes.* Drugs were identified using at least one prescription preceding the heart failure diagnosis date. Categories are mutually exclusive. Non-descript codes were labelled 'diabetes' without specifying Type 1 or 2. These codes were subjected to the type 1 and type 2 algorithms. If they were found to be potential type 1 patients using either algorithm they were excluded. *Algorithm taken from de Lusignan S, Khunti K, Belsey J, Hattersley A, van Vlymen J, Gallagher H, et al. A method of identifying and correcting miscoding, misclassification and misdiagnosis in diabetes: a pilot and validation study of routinely collected data. Diabet Med. 2010 Feb;27(2):203-9.

© 2019 Lawson CA et al. *JAMA Network Open.*

eFigure 3. Cumulative Incidence by Cause of Death

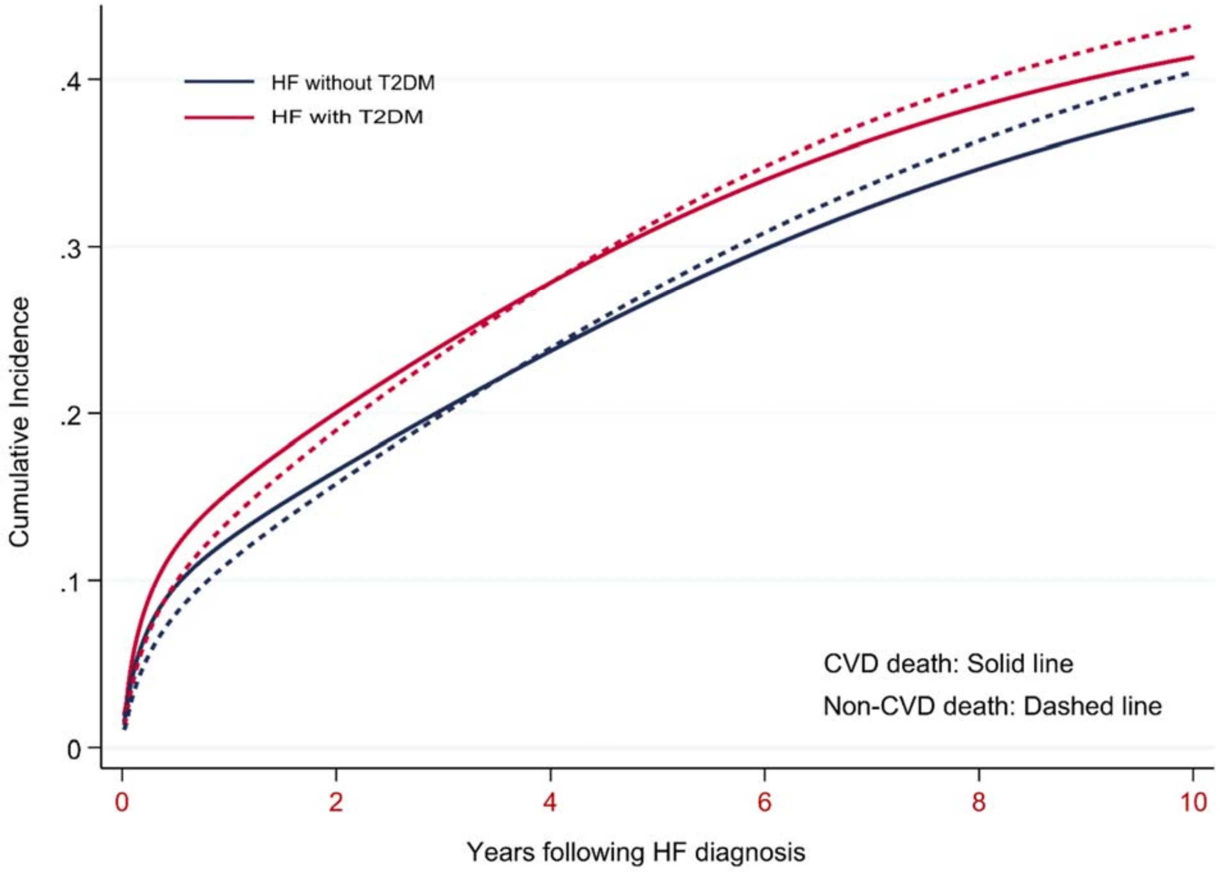

Cumulative incidence curves predicted at mean population age (78 years) at mid calendar time (2007).

© 2019 Lawson CA et al. *JAMA Network Open.*

eFigure 4. Trends in Risk Factors

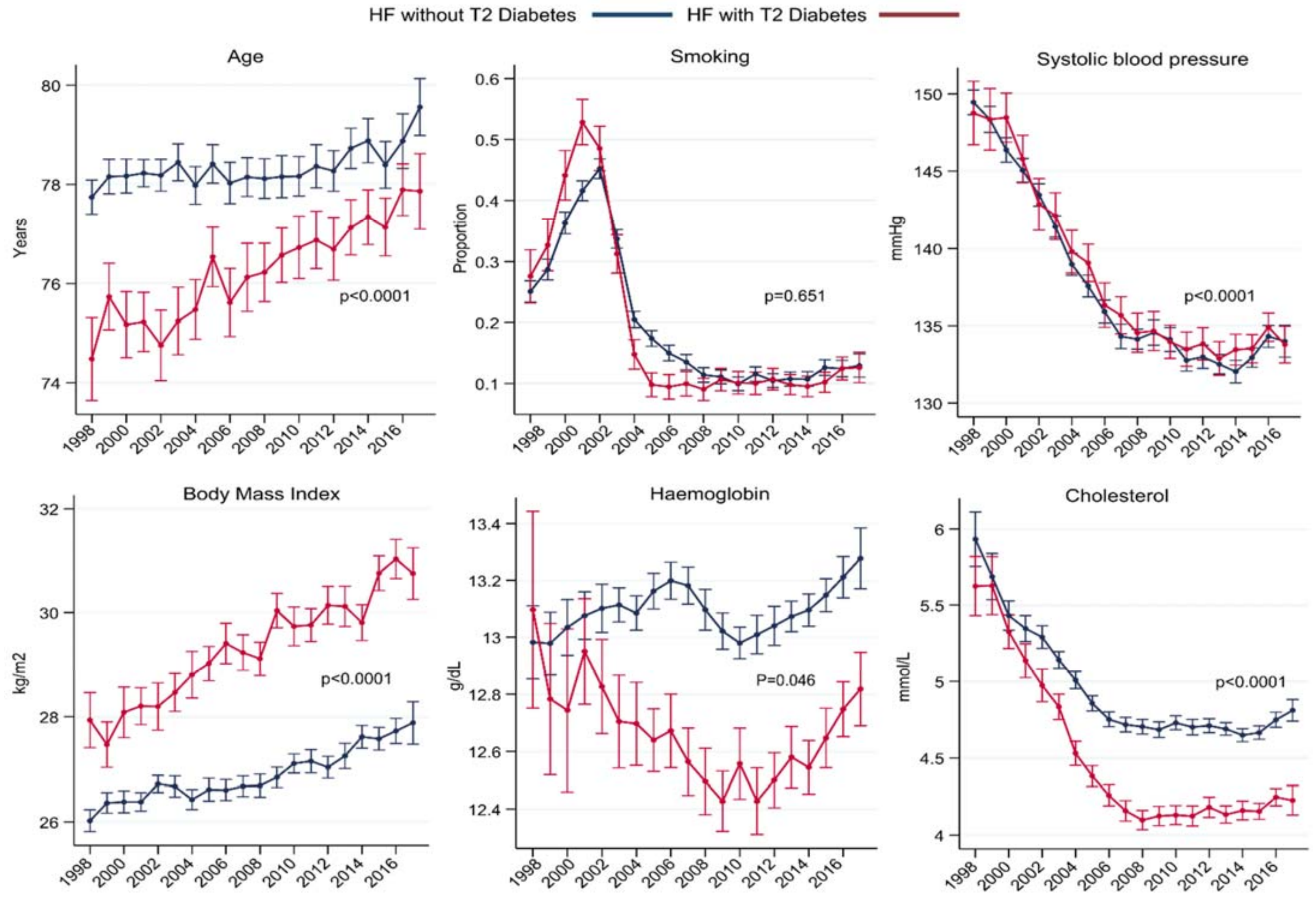

Estimated age-adjusted cardiovascular risk factors in patients with new heart failure by T2DM status and calendar year of HF diagnosis. Spikes indicate 95% CI. P-values test the difference in trends.

© 2019 Lawson CA et al. *JAMA Network Open.*

eFigure 5. Trends in Comorbidities at HF Diagnosis by Diabetes Status

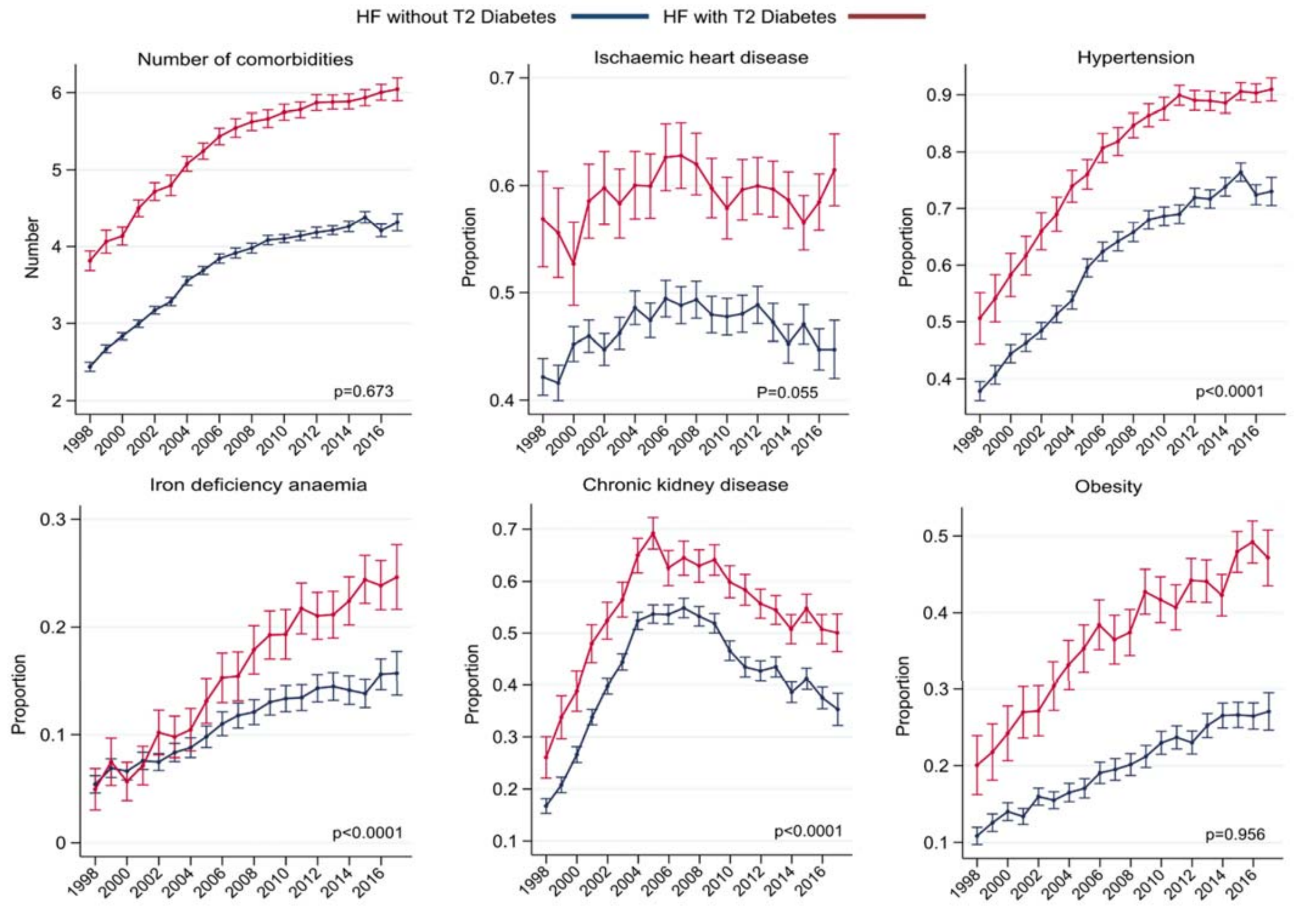

Estimated age-adjusted prevalence of comorbidities in patients with new heart failure by T2DM status and calendar year of HF diagnosis. Spikes indicate 95% CI. P-values test the difference in trends

© 2019 Lawson CA et al. *JAMA Network Open.*

eTable 1. Heart Failure Selection CPRD Code Set		
**Medcode**	**Read code**	**Read term**
2062	G58..00	Heart failure
1223	G58..11	Cardiac failure
398	G580.00	Congestive heart failure
2906	G580.11	Congestive cardiac failure
10079	G580.12	Right heart failure
10154	G580.13	Right ventricular failure
9524	G580.14	Biventricular failure
23707	G580000	Acute congestive heart failure
32671	G580100	Chronic congestive heart failure
27884	G580200	Decompensated cardiac failure
11424	G580300	Compensated cardiac failure
94870	G580400	Congestive heart failure due to valvular disease
884	G581.00	Left ventricular failure
23481	G581.11	Asthma - cardiac
43618	G581.12	Pulmonary oedema - acute
5942	G581.13	Impaired left ventricular function
5255	G581000	Acute left ventricular failure
27964	G582.00	Acute heart failure
101138	G583.00	Heart failure with normal ejection fraction
101137	G583.11	HFNEF - heart failure with normal ejection fraction
106897	G583.12	Heart failure with preserved ejection fraction
104275	G584.00	Right ventricular failure
4024	G58z.00	Heart failure NOS
12590	G58z.11	Weak heart
17278	G58z.12	Cardiac failure NOS
9913	1O1..00	Heart failure confirmed
22262	G1yz100	Rheumatic left ventricular failure
21837	G232.00	Hypertensive heart&renal dis wth (congestive) heart failure

© 2019 Lawson CA et al. *JAMA Network Open.*

eTable 2. Heart Failure Selection *ICD-10* Code Set

**ICD-10 Code**	**ICD-10 term**
I50	Heart failure
150.0	Congestive heart failure
I50.1	Left ventricular failure
I50.9	Heart failure, unspecified
I11.0	Hypertensive heart disease with (congestive) heart failure
I13.2	Hypertensive heart and renal disease with both (congestive) heart failure and renal failure
I13.0	Hypertensive heart and renal disease with (congestive) heart failure

© 2019 Lawson CA et al. *JAMA Network Open.*

eTable 3. Glucose-Lowering Medications	
Medication	**Number (%)**
None	8,514 (40.8)
Metformin	7,637 (36.6)
Sulphonylureas	6,021 (28.9)
Thiazolidinediones	1,168 (5.6)
Incretins	656 (3.2)
Other oral medications	232 (1.1)
Insulin	3,606 (17.3)
Summary categories	
None	8,514 (40.8)
Oral	8,738 (10.0)
Oral and Insulin	1,838 (2.1)
Insulin only	1,768 (2.0)

© 2019 Lawson CA et al. *JAMA Network Open.*

eTable 4. Missing Data

Characteristics	**No. Missing (%)**
Age	-
Gender	-
Socioeconomic status	0.2
Ethnicity	7.8
Prescribed drugs	0
Comorbidities	0
Smoking	5.9
Alcohol	14.1
BMI (kg/m^2)	14.8
Systolic BP (mm/Hg)	2.6
Total cholesterol (mmol/L)	32.9
Haemoglobin (g/dL)	24.6
eGFR (ml/min/m^2)	23.4

BMI, body mass index; **BP**, blood pressure;

eGFR, estimated glomerular filtration rate

© 2019 Lawson CA et al. *JAMA Network Open.*

eTable 5. Patient Characteristics at Diagnosis of Heart Failure by Diabetes Status[a]

Characteristic	No. (%)	
	No Diabetes (n = 66 851)	Type 2 Diabetes (m = 20 858)
Age, median (IQR), y	80.0 (72.0-86.0)	78.0 (70.0-84.0)
Women	33 768 (50.5)	9405 (45.1)
Race/ethnicity		
White	59 577 (91.9)	18 634 (90.4)
South Asian	597 (0.9)	712 (3.5)
Black	360 (0.6)	310 (1.5)
Other[b]	465 (0.7)	245 (1.2)
Socioeconomic status		
Most affluent quintile	13 042 (19.5)	3315 (15.9)
Most deprived quintile	10 659 (16.0)	4086 (19.6)
Hospital diagnosis	25 525 (38.2)	10 569 (50.7)
Prescribed drug therapy		
β-blocker	19 134 (28.6)	7287 (34.9)
ACE inhibitor	23 374 (35.0)	9358 (44.9)
ARB	5927 (8.9)	3142 (15.1)
AA	4036 (6.0)	1682 (8.1)
Aspirin	24 266 (36.3)	9288 (44.5)
Loop diuretic	32 111 (48.0)	10 821 (51.9)
Comorbidities		
No., mean (SD)	3.7 (1.9)	5.4 (1.9)
IHD	31 167 (46.6)	12 370 (59.3)
MI	16 273 (24.3)	6924 (33.2)
AF	26 409 (39.5)	8321 (39.9)
Hypertension	39 629 (59.3)	16 774 (80.4)
Stroke	7378 (11.0)	3207 (15.4)
Anemia	7550 (11.3)	3539 (17.0)
Obesity	13 374 (20.0)	8622 (41.3)
CKD	26 881 (55.2)	10 638 (57.4)
COPD	12 091 (18.1)	4266 (20.5)

© 2019 Lawson CA et al. *JAMA Network Open.*

Asthma	11 945 (17.9)	4451 (21.3)
Depression	14 582 (21.8)	5291 (25.4)
Osteoarthritis	24 351 (36.4)	8079 (38.7)
Cancer	15 648 (23.4)	4751 (22.8)
Dementia	3073 (4.6)	896 (4.3)
Active smoking status	13 382 (21.5)	3822 (18.7)
Active alcohol use	41 181 (73.2)	12 220 (63.9)
BMI, median (IQR)	26.2 (23.1-29.8)	29.0 (25.3-33.6)
Systolic BP, mean (SD), mm Hg	138.2 (21.9)	136.9 (20.9)
Total cholesterol, median (IQR), mg/dL	181.5 (154.4-216.2)	162.2 (135.1-193.1)
Hemoglobin, mean (SD), g/dL	13.1 (1.9)	12.7 (1.9)
eGFR, mean (SD), ml/min/m^2	57.9 (20.4)	56.6 (21.9)

Abbreviations: AA, aldosterone antagonist; ACE, angiotensin-converting enzyme; AF, atrial fibrillation; ARB, angiotensin receptor blocker; BMI, body mass index (calculated as weight in kilograms divided by height in meters squared); BP, blood pressure; CKD, chronic kidney disease; COPD, chronic obstructive pulmonary disease; eGFR, estimated glomerular filtration rate, HF, heart failure; IHD, ischemic heart disease; IQR, interquartile range; MI, myocardial infarction.

SI conversion factors: To convert total cholesterol to mmol/L, multiply by 0.0259; and hemoglobin to g/L, multiply by 10.0.

[a]Except for AF, cancer, and dementia, all differences were significant (ie, $P < .001$).

[b]Includes patients coded as mixed race, other race/ethnicity, or unknown.

© 2019 Lawson CA et al. *JAMA Network Open.*

**eTable 6.** Predicted Rates of Death During the First and Subsequent Years Following HF Diagnosis, by Type 2 Diabetes Status and Calendar Year								
	Deaths in first month	Predicted rate per 100 person-years (95% CI)			P Interaction [a]	Annual average percent change in rates (95% CI) and any significant change in trend slope[b]		
	N(%)	Overall	1998-2001	2014-2017			Year of change	
	**Rates during the first year following heart failure diagnosis in those who survived the first month**							
HF without diabetes	8,882 (13.3)	22.4 (21.7, 23.1)	22.9 (21.8 - 24.0)	21.0 (19.9 - 22.1)		-0.7 (-1.2, -0.2)	N/A	-0.7 (-1.2, -0.2)
HF with T2DM	2,629 (12.6)	28.5 (27.5, 30.0)	32.3 (29.0 - 35.6)	26.8 (24.9 - 28.7)	<0.0001	-1.4 (-1.8, -0.9)	N/A	-1.4 (-1.8, -0.9)
	**Rates after the first year following heart failure diagnosis in survivors of the first year**							
	Deaths in first year	Predicted rate per 100 person-years (95% CI)			P Interaction [c]	Slope change Annual percent change before and after break (95% CI)[e]		
		Overall	1998-2001	2014-2017				
HF without diabetes	20,229 (30.3)	14.9 (14.7, 15.1)	16.7 (16.4 - 17.1)	13.5 (13.0 - 14.1)		-1.6 (-1.8, -1.4)	N/A	-1.6 (-1.8, -1.4)
HF with T2DM	6,458 (31.0)	20.3 (19.1, 20.7)	23.8 (22.7 - 24.9)	17.3 (16.4 - 18.3)	0.013	-2.3 (-2.7, -1.9)	N/A	-2.3 (-2.7, -1.9)

HF, heart failure**; T2DM**, type 2 diabetes mellitus; **CI**, confidence interval

a P value for the difference in trend lines between groups. Estimated by fitting an interaction term between calendar year as a continuous variable and T2DM status in the negative binomial models also containing age.

b Average annual percentage change in rates (per 100 person years) for each increasing year of diagnosis. Any significant change in the trend line for rates was estimated using Joinpoint regression. Where present, rates are reported before and after the year of change.

© 2019 Lawson CA et al. *JAMA Network Open.*

eTable 7. **Age-Standardized Predicted Mortality Risk by Calendar Time**

	1-year			3-year			5-year		
Group	Overall	1998-2001	2012-2015	Overall	1998-2001	2012-2015	Overall	1998-2001	2012-2015
HF without diabetes	29.2 (29.0, 29.5)	30.9 (30.5,31.4)	27.0 (26.5,27.5)	46.7 (46.3, 47.0)	48.8 (48.3,49.4)	43.7 (43.1,44.4)	59.2 (58.9, 59.6)	61.5 (60.9,62.0)	56.1 (55.4,56.8)
HF with T2DM	35.6 (35.1, 36.1)	37.5 (36.8,38.1)	33.0 (32.4,33.6)	54.6 (54.0, 55.1)	56.8 (56.0,57.5)	51.4 (50.7,52.2)	67.2 (66.7, 67.7)	69.3 (68.6,70.0)	64.1 (63.3,64.9)

T2DM; type 2 diabetes. Overall rates were also standardised for calendar year.

© 2019 Lawson CA et al. *JAMA Network Open.*

eTable 8. **Associations With Hospital Admission and Death in Complete Case Analysis**

	All	CVD	Non-CVD
**Group**	Adjusted	Adjusted	Adjusted
**Hospital Admissions (IRR with 95% CI)**			
HF without diabetes (ref)	1.0	1.0	1.0
HF with type 2 diabetes	1.25 (1.23, 1.27)	1.21 (1.17, 1.25)	1.27 (1.24, 1.30)
**Death (HR with 95% CI)**			
HF without diabetes (ref)	1.0	1.0	1.0
HF with type 2 diabetes	1.14 (1.10, 1.18)	1.08 (1.03, 1.13)	1.21 (1.15, 1.28)

CVD; Cardiovascular**, IRR**; Incidence rate ratio, **CI**; Confidence interval, **HR**; Hazard ratio
Adjustment included age, gender, socioeconomic status, ethnicity, place of diagnosis, calendar year, beta-blocker, angiotensin converting enzyme inhibitor, angiotensin receptor blocker; aldosterone antagonist, aspirin, loop diuretic, number of comorbidities, ischaemic heart disease, myocardial infarction, atrial fibrillation, hypertension, stroke, anaemia, obesity, chronic kidney disease, chronic obstructive pulmonary disease, asthma, depression, osteoarthritis, cancer and dementia, current smoking, current alcohol drinker, body mass index, systolic blood pressure, cholesterol, haemoglobin and estimated glomerular filtration rate.

© 2019 Lawson CA et al. *JAMA Network Open.*
